# Spatial and Temporal Dynamics in Air Pollution Exposure Assessment

**DOI:** 10.3390/ijerph15030558

**Published:** 2018-03-20

**Authors:** Daniela Dias, Oxana Tchepel

**Affiliations:** Department of Civil Engineering, CITTA, University of Coimbra, Rua Luís Reis Santos, Polo II, 3030-788 Coimbra, Portugal; oxana@uc.pt

**Keywords:** personal exposure, air pollution, urban areas, spatial and temporal dynamics, numerical modelling, monitoring

## Abstract

Analyzing individual exposure in urban areas offers several challenges where both the individual’s activities and air pollution levels demonstrate a large degree of spatial and temporal dynamics. This review article discusses the concepts, key elements, current developments in assessing personal exposure to urban air pollution (seventy-two studies reviewed) and respective advantages and disadvantages. A new conceptual structure to organize personal exposure assessment methods is proposed according to two classification criteria: (i) spatial-temporal variations of individuals’ activities (point-fixed or trajectory based) and (ii) characterization of air quality (variable or uniform). This review suggests that the spatial and temporal variability of urban air pollution levels in combination with indoor exposures and individual’s time-activity patterns are key elements of personal exposure assessment. In the literature review, the majority of revised studies (44 studies) indicate that the trajectory based with variable air quality approach provides a promising framework for tackling the important question of inter- and intra-variability of individual exposure. However, future quantitative comparison between the different approaches should be performed, and the selection of the most appropriate approach for exposure quantification should take into account the purpose of the health study. This review provides a structured basis for the intercomparing of different methodologies and to make their advantages and limitations more transparent in addressing specific research objectives.

## 1. Introduction

Air pollution is considered the world’s largest single environmental health risk, contributing to around 7 million premature deaths worldwide, as reported by the World Health Organization (WHO), and urban citizens are particularly affected [1,2]. Although emissions of many air pollutants have decreased, the European Environment Agency estimates that about 30% of Europe’s urban population is still exposed to air pollution concentrations exceeding the EU air quality limits set to protect human health [3]. According to the Organisation for Economic Co-operation and Development, by 2050, air pollution is anticipated to become the biggest environmental cause of mortality worldwide, overtaking the lack of clean water and poor sanitation [4]. The evidence for the adverse health effects from exposure to air pollution is robust, even though there are still knowledge gaps regarding the exact mechanisms by which air pollutants affect human health (including the effects of pollutant mixtures), and which pollutants should be tackled with priority [5,6].

Given the need for a better understanding on the potential impact of urban air pollution on human health [7], exposure assessment presents an important tool to describe and determine quantitatively the amount of air pollutants which individuals are actually exposed to. Consequently, human exposure assessment composes an essential and critical component for health impact assessment (HIA) and for the design of air pollution control policies.

Over the past decades, numerous methods for assessing human exposure levels to air pollution have been used by several studies focusing on the links between air pollution and health, with the goal of estimating exposure at individual level within an entire study population. However, the main criticism of these studies relates to the quality of exposure data and its relationship with true personal exposures in the study area. An inaccurate quantification of true exposure leads to considerable uncertainty in health risk estimates [8,9]. Several approaches do not take into account all exposure situations that a person experiences in their daily life for the exposure assessment, and conclusions about the total exposure on an individual level are therefore not addressed.

The challenge, however, is that individual exposure to air pollution in urban areas results from a dynamic process and multifaceted iterations between the human being and urban air, depending both on the spatial-temporal dynamics of air pollution concentrations and the individual’s activities. Consequently, individuals have their very own unique personal exposure to air pollution during their daily life, occurring both in indoor and outdoor environments, and thus the quantifying process is not straightforward.

The review presented in this paper is focused on the concepts, key elements and methods available and required to quantify personal exposure at the spatial and temporal scale, imposed by the behavior of individuals in urban areas. Crucial questions such as “How should personal exposure to air pollution be defined?”, “What are the key elements of personal exposure assessment?” and “How can personal exposure to air pollution be quantified?” are addressed in this review. Moreover, a new conceptualization of personal exposure assessment based on two classification criteria (characterization of air quality and characterization of individual’s activities) is proposed and presented.

## 2. How Should Personal Exposure to Urban Air Pollution Be Defined?

In 2004, the glossary of the International Programme on Chemical Safety was adopted as the official glossary of the International Society of Exposure Analysis [10] defining exposure as the “concentration or amount of a particular agent that reaches a target organism, system, or (sub)population in a specific frequency for a defined duration” [11,12,13]. However, the word “exposure” has different meanings in different contexts. Reviewing the complex and varied fields of exposure assessment, risk assessment, environmental health, epidemiology and toxicology makes it possible to find several definitions of exposure, depending on the needs and objectives of the different research areas (e.g., [14,15,16,17,18]).

The increasing evidence that each individual is subject to his/her own individual exposure due to his daily activity patterns highlights that exposure to air pollution is not a static phenomenon, making a clear distinction between population exposure and personal exposure [19,20,21,22,23,24].

Exposure is quantified as a function of concentration and time and can be represented by several time-exposure metrics. Depending on the time of exposure, instantaneous, time-integrated and time-average exposure could be distinguished [19,25,26]. Instantaneous exposure is the exposure at an instant in time and is expressed in the same unit as the concentration (e.g., μg·m^−3^), while time-integrated exposure is the integral of instantaneous exposures over the duration of exposure (units: ppm·h or μg·m^−3^·h) (Equation (1)) [27]. It is important to mention that an “instantaneous” exposure measurement depends on the response time of the instruments or the sampling volume that should be specified in studies addressing this exposure metric:

(1) $$E_{i}\;=\;\int_{t_{1}}^{t_{2}}C_{i}\;(x,y,z,t)\;dt$$

where *E_i_* is the time-integrated exposure experienced by the individual *i*, *C_i_* (*x*,*y*,*z*,*t*) is the concentration occurring at a particular point occupied by the individual *i* at time *t* and spatial coordinate (*x*,*y*,*z*), corresponding *t*_1_ and *t*_2_ to the starting and ending times of the exposure event, respectively. This type of exposure can be estimated through measurements (e.g., via personal air monitors) that usually provide incremental data on exposure [28].

Other possible formulations of exposure that depend on the time of exposure include time-averaged exposure and peak exposure (units: ppm or μg/m^3^) [27]. Time-averaged exposure is determined by dividing the time-integrated exposure by the duration of the exposure (*t*_2_ − *t*_1_) (Equation (2). This can be a useful formulation for many environmental applications (e.g., daily average exposure) and is relevant for both acute and chronic health effects. The peak exposure is usually relevant for short-term exposure and acute toxic effects [29]. The time period to be considered in the exposure time profile should be defined under the scope of the exposure analysis (e.g., a biologically relevant time period):

(2) $$E_{i}\;=\;\frac{1}{t_{2}-t_{1}}\int_{t_{1}}^{t_{2}}C_{i}\;(x,y,z,t)\;dt$$

There is a clear distinction between air pollution concentration and exposure, which requires a contact of air pollution with an individual. High air pollution concentrations do not necessarily result in high exposure. The concentration of a specific air pollutant is subject to high variability in space and in time depending on variations of emission sources, meteorology, land use and terrain [30,31]. In addition to air pollution concentrations, the exposure depends on time-activity patterns of individuals [22,32,33,34,35].

Within this context, individual exposure to air pollution should be defined as the real concentration of air pollutant breathed in by the individual at a particular time and place, and it does not only arise from the pollutant concentration in the environment to which the individual is exposed but is also determined by the amount of time spent in that environment.

## 3. What Are the Key Elements of Personal Exposure Assessment?

Despite significant improvement in the quality of exposure assessments over the past 20-year history of the HIA, admittedly, there are several key components that should be considered for personal exposure assessment to urban air pollution [36,37,38], as described below.

### 3.1. Spatial and Temporal Variability of Urban Air Pollution

An important component of personal exposure assessment is a better understanding of spatial and temporal variability in pollutant concentrations. The dynamics in emissions namely from road transport (e.g., activity patterns such as the morning rush hour leading to peaks in traffic-related pollution) are one of the factors leading to the significant variation of air pollutants concentrations in cities.

After being released by emission sources, air pollutants can be transported and transformed through a number of physical and chemical processes at a range of spatial and temporal scales. In urban areas, the transport and dilution of air pollutants are affected by meteorological conditions and local conditions (e.g., urban form, built-up areas, street canyons, road networks). The presence of high buildings on both sides of the road creates a “street canyon”, which reduces the dispersion of the emitted pollutants from traffic sources and can lead to significantly higher concentrations locally. There is also evidence to suggest that air pollution concentrations decrease to background levels behind a row of uninterrupted buildings [29]. Various monitoring studies have suggested that in cities, strong variability of air pollution may occur over small distances (<100 m) [39]. Thus, air pollution data from a single monitoring station can only be considered representative of a rather small surrounding area. Such measurements are dramatically affected by the stations’ location, and do not adequately capture the spatial variability for pollutants with local sources [40].

Urban air is an umbrella concept, combining outdoor and indoor air. In addition to the significant temporal and spatial variability of outdoor concentrations, scientific evidence has shown that indoor environment plays a significant role in personal exposure to air pollution, where urban populations spend large fractions of their time throughout life [33,41,42]. It is known that most people in European cities spend on average about 80–90% of their time indoors, 1–7% in a vehicle, and only 2–7% outdoors [43,44]. Thus, indoor spaces represent important microenvironments when addressing personal exposure to air pollution. Moreover, several findings indicate that indoor concentrations are typically higher than the respective ambient levels [45,46]. Nevertheless, it is important to note that sampling indoor air is not enough to understand personal exposure and it has been demonstrated that personal exposure does not correlate well with measurements of indoor concentrations [19].

### 3.2. Spatial and Temporal Dynamics of Individual’s Activities

Human behavior and use of time is referred to as the time-activity pattern of an individual, and are strongly linked to various personal characteristics including age, gender, education, income and employment status [47]. Urban areas, where around 75% of the European population currently lives, are complex systems comprised of individuals characterized by different behavioral patterns [48,49]. The urban environment accommodates services, employment opportunities and other facilities, where individuals may conduct their desired activities, which affects their mobility significantly.

In the context of human exposure, an understanding of human mobility patterns is crucial, as these strongly influence the assessment accuracy of actual human exposure to air pollution [22,50,51,52]. Analyzing time-activity patterns for personal exposure assessment may indicate the distribution of time among activities and the factors that influence the degree of media contamination in the activities, and reflect the duration of contact during the activities [53]. Also, there is an inter- and intra-variability of individual’s activities, which has implications for the use of time-activity data in exposure assessment. Several studies on time–activity patterns used in epidemiologic studies are available [54,55]. The information needed in such studies include location of the activity, the period of time when the activity took place (e.g., time of day, phase in life), and the duration of the activity.

**Figure 1:**
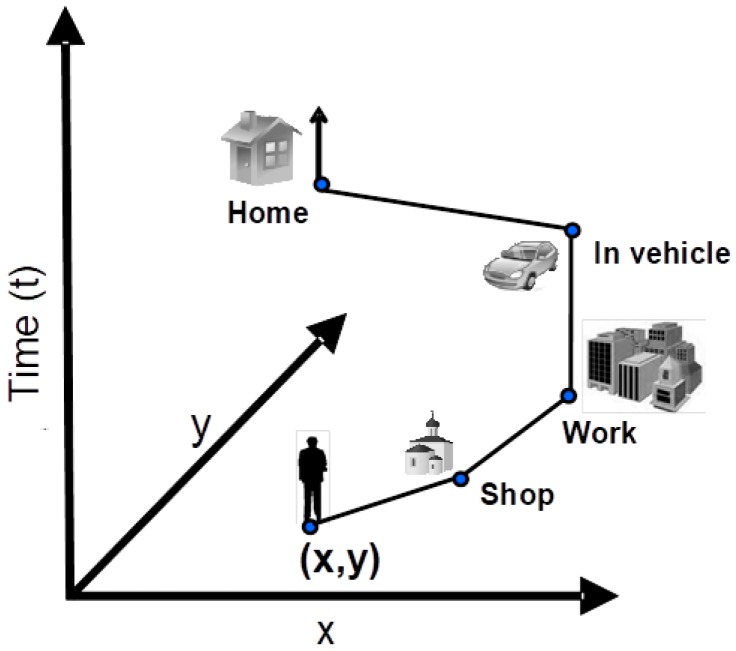
Trajectory of an individual in space (x, y) and time (t).

International studies focusing on exposure to air pollution, such as Total Exposure Assessment Methodology (TEAM) studies [56], the National Human Activity Pattern Survey (NHAPS) [57] and the Population Exposure to Air Pollutants in Europe (PEOPLE) project [58] relied on diary-based instruments (e.g., time-activity diaries (TADs), questionnaires, California Household Travel Survey, National Household Travel Survey, etc.) to categorize the environments where exposure occurred and sources of air pollutants, and to derive information on the temporal sequencing of human activities during the study period. However, such time-activity information does not account for the movement of the individual and mostly lacks the exact “activity-space” where a specific activity is executed by the individual [59,60] and consequently, the sequence of exposure events is not considered (Figure 1).

To overcome some of the uncertainties related to the human mobility during the exposure assessment period [61], the availability of GPS for human tracking presents an enormous opportunity for improving our understanding of how time-activity patterns can influence individual exposure and subsequent health effects. GPS is a freely accessible and promising technology which may answer crucial questions such as “Where are individuals located during their daily activities?” by monitoring individuals’ real-time geographic positions, thus providing new insights in the field of personal exposure assessment to air pollution in urban areas.

Studying and predicting mobility patterns of individuals using cell phones with built-in GPS receivers is an emerging field [62,63]. GPS-equipped mobile phones can record the latitude-longitude position of individuals at each moment, offering many advantages over traditional time-location analysis, such as high temporal resolution, and ensure a minimum reporting burden for participants [59,64]. However, GPS is not a standalone tool used to determine time-activity locations, such a commuting, indoor or outdoor locations, since it can only provide information on the path that a moving individual follows through space as a function of time, i.e., GPS trajectory [59,65,66]. Significant uncertainties associated with the processing and classifying of GPS trajectories is one of challenges of the exposure studies [65].

## 4. Spatiotemporal Personal Exposure Assessment: What Are the Methods Available?

Under a traditional perspective, the evaluation of human exposure to air pollution can be carried out under a: (i) direct approach or (ii) indirect approach. With the direct approach, exposure levels are measured at the individual level, based on personal monitoring or using biological markers. With the indirect approach, exposure levels are usually estimated or modelled based on ambient measurements, exposure modelling and surveys [19]. In addition, according to USEPA’s Guidelines for Exposure Assessment, exposure can be quantified in three different ways: (i) point-of-contact measurement or personal monitoring in which exposure can be measured at the point of contact (the external boundary of the body) while it is taking place, (ii) reconstruction of internal exposure through the use of internal indicators (biomarkers, body burden, excretion levels, etc.) after the exposure has taken place and (iii) the exposure scenario evaluation in which the exposure is estimated considering hypothetical but plausible scenarios to analyze the concentration and contact time, through the use of models [67].

Nevertheless, under a traditional framework, major air pollution exposure assessments assume a static location for the individual. However, the implementation of comprehensive approaches to address exposure accounting for individual’s activities in space and time is required [68,69], and has been identified as a priority area in exposure research [70]. This new context of exposure has emerged strongly supported by the recent development of geo-spatial technologies [71,72,73], moving from a static assessment to dynamic personal exposure assessment.

**Figure 2:**
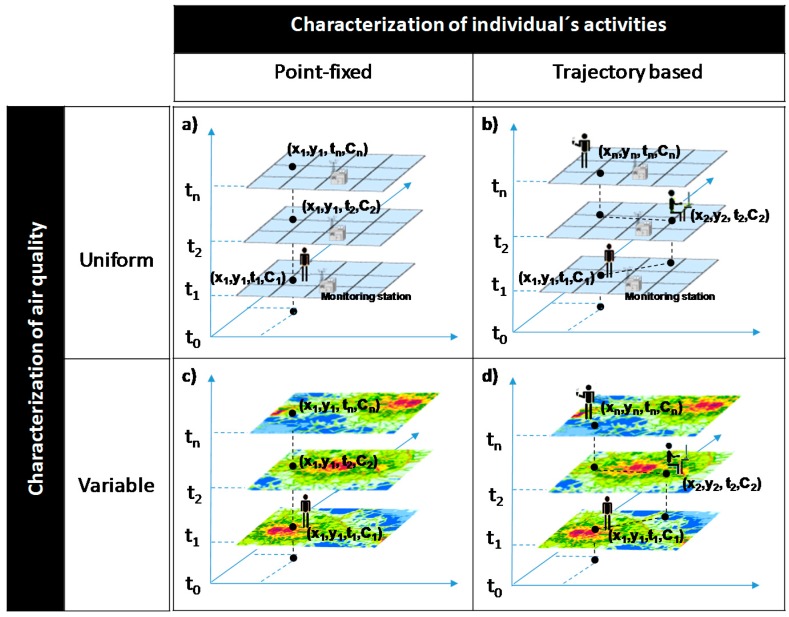
Combination of the classification criteria for personal exposure assessment: (**a**) individual point-fixed activities and uniform air quality approach; (**b**) trajectory based and uniform air quality approach; (**c**) individual point-fixed activities and space-variable air quality approach; (**d**) trajectory based and space-variable air quality approach.

Given recent advancements in the field of personal exposure assessment, there is an important need to classify methods for assessing personal exposure taking into account the spatial and temporal dynamics of exposure. Therefore, a novel conceptualization of personal exposure assessment is proposed and used in this study based on two classification criteria: (i) the characterization of individual daily activities location (point-fixed or trajectory based) and (ii) the characterization of air quality (variable or uniform). The proposed classification scheme for personal exposure assessment methods is depicted in Figure 2.

**Figure 3:**
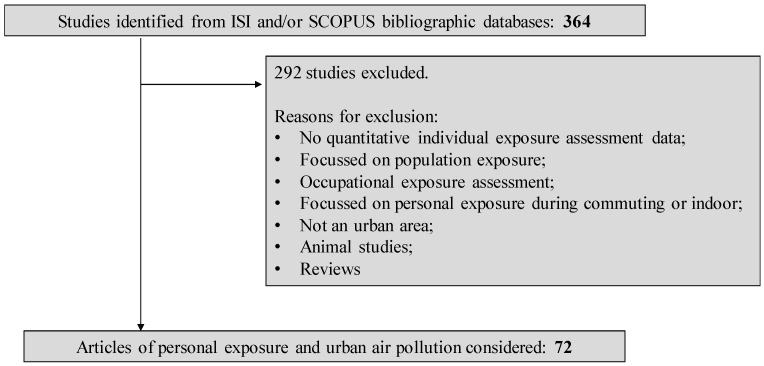
Summary of the article selection process.

A literature review on various approaches currently available to quantify individual-level exposure to urban air pollution was conducted based on journal articles published in English from 2006 to June 2017 and indexed by ISI and/or SCOPUS. The search was performed considering the combination of the following search terms: “personal exposure”, “individual exposure”, “urban air pollution”, “urban area”, and “air pollution”. Three hundred and sixty four articles were identified from Web of Science database. Among these, two hundred and ninety two studies were excluded since: no quantitative information on individual exposure were provided; they were only focused on population-level exposure; the main objective was occupational exposure assessment; personal exposure assessment was only performed during commuting or staying indoors; total personal exposure was not addressed; they were not focused on urban areas; they were reviews or animal studies (Figure 3).

From the search performed, 72 studies were selected for full-text review. Table 1 provides a summary of each study. This review article is intended to provide concise and critical updates on the methods currently used to capture air pollution dynamics and daily activity patterns in exposure assessment. Despite its acknowledged importance, a summary of the quantitative outcomes from the individual exposure studies is not in the scope of this work*.*

### 4.1. Personal Exposure Assessment Based on Point-Fixed Activities and Uniform Air Quality

Under a point-fixed and uniform air quality approach, exposure levels are examined by subdividing a study area into homogeneous sub-areas, based usually on census data and assigning individual daily activities to the residence location. This is a standard and static approach, where the personal exposure concentration is simply deduced by the air pollutant concentrations in ambient (outdoor) air from background monitoring stations with no spatial variation. Therefore, it is assumed that monitoring data is representative for a large area and all individuals living within this area are equally exposed.

From the literature review, many studies assumed point-fixed activities and uniform distribution of air pollutant concentrations. However, since 2006, only eleven studies relying on such approach were identified [68,74,75,76,77,78,79,80,81,82,83]. Air concentration measurements from nearest central-site monitoring station and a fixed location of the individual, typically residential address [68,75,76,78,79,80,81,82] or school address [74,77,83], are considered in these studies. From the performed review, Gao et al. [83] is the most recent study that uses measurements of air pollutant concentrations to examine the relationship between long-term exposure to air pollution and respiratory morbidities in Chinese children [83]. Annual means of PM_10_, SO_2_, NO_2_ and O_3_ from urban air monitoring stations closest to the primary schools were used to estimate the individual exposure of school children, assigning individual daily activities to the school location. Only the primary schools located within 1 km of the local air monitoring station were included in the study. In order to reduce exposure misclassification, the authors indicate that only students who had been currently living in the district where their school was located for more than 12 consecutive months prior to the study were selected [83]. Therefore, it was assumed that children spent the majority of their daily time in school and all children studying in the same school are equally exposed.

Overall, these point-fixed location/uniform air quality studies do not consider the significant degree of variability over space and time that characterizes both an individual’s activities and the urban air pollution that they are exposed to. In such studies, the same measured air pollution concentration is assigned to people occupying the same defined areas (e.g., city, urban agglomeration), assuming a static place/location for the individual. However, measurements from central-site monitors often do not adequately capture the greater spatial and temporal variability of pollutant concentrations within an urban area, which may result in an underestimation of the inter- and intra-variability of personal exposure within the study population. Also, central-site monitors do not account for exposures in different microenvironments (e.g., indoors and in-vehicle) where pollutant infiltration and indoor sources can substantially impact total exposures. Residential address is generally used as the surrogate for the personal exposure, when in fact a high percentage of an individual’s exposure can occur from relatively short periods of time spent in high-polluted microenvironments (e.g., indoors and in-vehicle) where pollutant infiltration and indoor sources can substantially impact total exposures, compared with the data at centrally located air quality monitoring stations [22,34,84,85].

In this context, air quality measurements should be used carefully in the quantification of personal exposure since there is a potential for exposure error and a resulting bias (e.g., underestimation of relative risks) when solely depending on ambient monitors to characterize exposure [22,53,86]. Despite the low cost of implementation, the main issue of studies that assess personal exposure using point-fixed activities and uniform air quality is that the inability to account for small-scale spatial variability can lead to significant exposure misclassification as such personal exposure assessment approach is unable to capture the spatial variation of air pollution within urban areas, with the intra-urban variation often greater than inter-urban variation. Also, individual time-activity patterns, such as time spent indoors vs. outdoors and time spent at work, home or school, are blurred by the use of this approach, which considers uniform exposure over an area for a given time period.

### 4.2. Personal Exposure Assessment Based on Point-Fixed Activities and Variable Air Quality

The point-fixed location/variable air quality approach for determining personal exposure, is focused on characterising the spatial and temporal variability in pollutant concentrations during the day, while daily activities are disregarded. Air pollutant concentration fields are characterised based on modelling techniques able to provide outdoor concentrations with high resolution in time and space. One important application is extending observations spatially in order to reduce exposure errors and uncertainties that arise from the limited spatial coverage of current routine monitoring networks in urban areas. However, this approach does not account for personal trajectory, and assigns individual daily activities to a point-located position, such as residential address.

From the literature review, 13 studies assessing individual exposure based on a point-fixed and variable air quality approach were identified. Under this approach, personal exposure is often estimated at the individual’s residential address by using air quality concentrations generated by Land Use Regression (LUR) techniques [87,88,89,90,91,92,93,94] and by air quality models [95,96,97,98]. In addition, one exposure study using geographic information systems (GIS)-based interpolation method to approximate outdoor concentrations near communities was identified [99].

LUR modelling takes advantage of GIS-based information on land-use and source proximity or characteristics (e.g., traffic volume) in a given modelling domain to create air pollutant concentration fields together with measured pollutant concentrations. Recent applications have incorporated physically based factors such as meteorology in an attempt to improve estimates (e.g., [94]). Though LUR models offer improved spatial resolution, they still may not capture a fine enough spatial resolution to predict individual exposure within urban areas (e.g., [87,88,92]).

Air quality modelling has been used to estimate air pollutant concentrations as a surrogate of exposure. As previously reviewed by Zou [100], such models help in determining the most reliable exposure simulation results. Air quality models estimate pollutant concentration profiles over space by applying mathematical formulations of chemical and physical processes to site specific input data on source emission and meteorology. From the analyzed studies, the various air quality models applied appeared to increase the spatiotemporal variability of ambient concentrations of pollutants when compared to the use of central-site monitoring data alone, especially for pollutants produced by local sources (e.g., [96,97]). Moreover, combining regional, urban and local-scale dispersion modelling provided a full spatiotemporal coverage of study areas as opposed to the limited point locations provided by ambient monitoring. The improved spatial resolution of air quality models had noticeable impacts on some epidemiologic estimates of health effects (e.g., [98]). Although air quality models are a promising tool to personal exposure assessment by characterizing the air pollution levels required to quantify exposure at the individual level, a significant uncertainty exists in constructing the exposure determination on outdoor levels at the residential address only, ignoring the contribution of other microenvironments to individual exposure (e.g., [96]).

Overall, the advantage of conducting personal exposure assessment based on point-fixed activities and variable air quality is their ability to provide air pollutant concentrations at very fine spatial resolution, capturing its spatial and temporal variation within urban areas. Also, it can be used to assess time periods from hourly to annual averages. However, this approach does not account for spatial and variation of individual time-activity patterns to assess personal exposure, and assigns individual daily activities to a point-located position.

### 4.3. Personal Exposure Assessment Based on Individual’s Trajectory and Uniform Air Quality

Under a trajectory based exposure approach with uniform air quality, spatial-temporal variations of an individual’s activities are considered for personal exposure assessment. In this case, the individual’s location and time spent is addressed by distinguishing several microenvironments, such as home and workplace, and by identifying the nearest pollution monitor to these locations.

From the performed literature review, four studies which estimate personal exposure using the individual’s trajectory/uniform air quality approach were identified. Under this approach, personal exposure is mainly assessed by using microenvironmental concentrations estimated using a mass-balance indoor model and the closest air quality monitoring station as a proxy for outdoor concentration [55,101,102,103]. In such studies, microenvironments are differentiated (e.g., home, school, others indoors) in terms of time spent in these locations based on time–activity data (e.g., time-weighted factors). The contributions of time spent during commuting is often ignored under this approach.

In an attempt to improve personal exposure estimates, one of the selected studies investigated the potential of using a complex modelling tool (pCNEM) to generate personal exposures, and compared the resulting associations with the concentration-response function (CRFs) estimated using routinely collected ambient concentrations [101]. The pCNEM model uses a complex stochastic process that follows the randomly selected individual in their activities over the period of the simulation based on time–activity databases (i.e., NHAPS). The individual’s location is addressed by distinguishing between home and workplace and by identifying the districts that are associated with the nearest air quality monitor. According to the authors, the efforts undertaken to characterize the spatial-temporal variations of individual’s activities had a noticeable impact on the concentration-response function estimates. They also observed that individual exposures to PM_10_ were lower than the measured ambient concentrations [101]. Likewise, other studies found that peaks of ambient PM concentrations do not necessarily reflect peaks of exposure, since the timing and indoor concentration significantly affect the actual exposure (e.g., [103]). As evidenced by Lane et al., this occurs especially for near highway and employed participants [55].

The trajectory approach offers improved spatial resolution of individual’s activities. However, it doesn’t capture adequately the inter- and intra-variability of personal exposure if no spatial variation for air quality data is considered. Physick et al. [102] demonstrated that exposure estimates for NO_2_ based on nearest monitoring station are consistently higher than exposure measurements by about 15% at the near home location, and underestimated to about 7% if the monitoring station close to the working place is considered, assuming evening and daily hours, respectively.

### 4.4. Personal Exposure Assessment Based on Individual’s Trajectory and Variable Air Quality

Under a trajectory based exposure approach with variable air quality, personal exposure levels may be directly measured by personal monitoring, or estimated by spatial-temporally resolved exposure models combined with time-activity diaries or GPS data to describe the trajectory of an individual.

From the literature review, 44 studies assessing individual exposure based on an individual’s trajectory and variable air quality approach were identified. The majority of the analyzed studies use personal monitoring as the most reliable and accurate way of estimating the pollution levels that an individual is actually exposed to [20,24,104,105,106,107,108,109,110,111,112,113,114,115,116,117,118,119,120,121,122,123,124,125,126,127]. Personal monitoring assesses an individual’s exposure based on measuring the concentration of a pollutant, ideally within a person’s breathing zone for a defined time. A variety of active (i.e., pumped instruments) and passive devices (e.g., diffusion tubes) have been used in exposure assessment studies to monitor personal exposure to air pollution as closely as possible to the breathing zone.

GPS technology has been used successfully in personal exposure assessment to collect the individuals’ time-location information. Several personal exposure studies have used a well-designed integration of GPS devices with portable pollutant monitors to determine potential exposure at the individual level (e.g., [22,127,128,129,130,131,132]). Several personal monitoring systems are now emerging, using sensors developed specifically for the purpose of personal or high density network monitoring where air pollution levels can be measured and/or estimated at small spatial and temporal resolutions and then combined with information on mobility and physical activity of the person (e.g., [28,133]). The advantage of such approach is that the likely costs could be much lower than traditional personal monitoring. However, the performance of these low-cost, wearable or portable sensors needs to be adequately validated prior to their use in data collection and sharing on a large scale.

The strength of personal sampling is the quantification of real exposure values for the individuals followed. The drawback of this approach, however, is the high cost of implementation. Also, the temporal resolution is limited since this approach provides exposure data for the individual only at the time of sampling, thus limiting the usefulness of its value in estimating long-term exposure. In addition, poor compliance with personal sampler wearing protocols can create positive or negative biases in the reported exposure concentrations, depending on the proximity of the participant or the personal sampler to the pollutant source when the monitor was not worn as instructed.

Based on the performed literature review, exposure modelling has arisen as an additional method of trajectory based and variable exposure assessment able to address the magnitude of air pollutant concentration thoroughly breathed in by the individuals during their daily activity patterns [86,134,135,136,137,138,139,140,141]. Exposure models that combine ambient concentrations with microenvironmental and behavior factors have the potential to improve personal exposure estimates. Moreover, such models have the ability to investigate large populations, future scenarios, as well as reconstruct historical exposure by utilizing existing data from different source types.

Several personal exposure models based on a microenvironment approach, including hazardous air pollutant exposure model (HAPEM) [86], micro-environmental exposure model (MEEM) [136] and activity-based modeling framework for Black Carbon exposure assessment (AB^2^C) [139] are available. These models are designed to simulate the distribution of personal exposure by combining the time spent at visited microenvironments and the estimated pollutant concentrations (e.g., PM_10_, VOCs, etc.) in each microenvironment. Usually, such modelling frameworks combine microenvironmental concentrations, estimated as a combination of infiltrated outdoor air and indoor source emissions based on mass balance or empirical indoor/outdoor relationships, and time–activity databases.

The literature review indicates that there has been an increasing focus on using GPS technology to collect individual trajectory information to be used in combination with air quality modelling to estimate personal air pollution exposure levels in urban areas (e.g., [134,137,138,140]). One of the first attempts to use GPS technology in personal exposure modelling was performed by Jensen [134]. This modelling framework, named AIRGIS, estimates exposure in the home and workplace at the address level. Also, it includes a model for the estimation of exposure under transport provided by cell phones with built-in GPS receivers, which send location information by short message service to the AIRGIS tracking centre at twenty seconds intervals [134]. Despite that AIRGIS is addressing the most significant microenvironments, conclusions about the contribution of other indoor microenvironments (e.g., shopping, restaurant, etc.) and outdoor activities to the total individual exposure, are therefore not possible.

Recently, some comprehensive exposure modelling systems that provide both spatially and temporally resolved exposures have emerged [137,138,140,142]. Specifically, such studies estimate exposure by “following” the individual during their daily routines using GPS technology, instead of considering the typical microenvironments, thus providing a time-sequence of the exposure events. The improved spatial resolution of modelled air pollutant concentrations, combined with detailed individuals’ time-location information, had noticeable impacts on exposure estimation, evidencing good compliance with personal exposure samples [140]. These studies also show that the integration of smartphone momentary location tracking and air quality modelling provides a feasible and cost-effective way to assess personal exposures in space-time [142].

It is important to note that the GPS technology may provide a large amount of data describing individual trajectories. However, as mentioned previously (Section 3.2), there is a challenge to process and classify the spatio-temporal patterns from raw GPS data. From the revised manuscripts, Dias and Tchepel [138] implements an automatic processing of GPS data using data mining analysis to identify time-activity location in several microenvironments. According to the authors, the results indicate that this approach could be used to extract and to analyze the time-activity patterns required for the exposure assessment.

## 5. Conclusions

Air pollution has emerged as one of the major health problems in urban areas, with direct consequences for the urban citizens’ health. In this article, the current developments in assessing personal exposure to air pollution in the urban environment, as well as the methods available and respective advantages and disadvantages, were reviewed. Additionally, important exposure-related concepts and key elements required to understand the human exposure science were also discussed.

As evidenced by the performed literature review, personal exposure estimation is crucial in determining the relationship between air pollution and health effects, and it is the most accurate indicator of what an individual breathes, influenced not only by the pollutant concentration in the environment but also on the amount of time spent by the individual in that environment.

The poor correlations often observed between individual exposures and ambient air concentrations suggest that a set of factors other than ambient air (outdoor) may contribute to personal exposures. The spatial and temporal variability of urban air pollution levels in combination with indoor exposures and individual’s time-activity patterns are key elements to a proper assessment of personal exposure. Thus, it is clear that analyzing individual exposure in urban areas offers several challenges, where large spatial and temporal dynamics of individuals and air pollution levels are observed.

In reviewing the current state of knowledge for personal exposure assessment, an emerging context of exposure assessment recognizing the importance of the actual spatial and temporal scales on quantifying personal exposure to air pollution is identified. Also, this review is enriched by aggregating personal exposure assessment methods according to two classification criteria: (i) spatial-temporal variations of individual’s activities (point-fixed or trajectory based) and (ii) characterization of air quality (variable or uniform). A point-fixed/uniform exposure approach assumes ambient air quality values homogeneous for a specific area, while a trajectory based/variable air quality approach considers both individual time-activity patterns and air pollution concentration variability.

The literature review (72 selected studies) reveals that personal exposure assessment has progressed significantly over the past decade, from crude qualitative exposure estimates under a point-fixed and uniform air quality approach (11 studies) to today’s refined integrated methods based on an individual’s trajectory and variable air quality approach (44 studies), yielding more accurate quantitative exposure estimates at the individual level. The availability of global positioning system (GPS) facilitates the collection of an individual’s spatio-temporal trajectory, and can greatly improve the accuracy and spatio-temporal resolution of existing time activity surveys, as evidenced by Dons et al. [22].

Based on the performed literature review, the majority of studies (44 in 72 reviewed studies) indicate that the trajectory based with variable air quality approach is a promising methodology of exposure analysis to provide the inter- and intra-variability of individuals’ exposure levels. Such an approach is identified as one of the most effective alternative able to address high spatial and temporal variation in concentration levels, thereby allowing the analysis of sources and pathways in the exposure assessment process. However, future quantitative comparison between the different approaches should be performed in order to avoid implementation of costly methods with little benefit. Moreover, the selection of the most appropriate approach should take into account the purpose of the health study and related factors, including the exposure duration (short-term or long-term), the health effects analyzed (acute or chronic), the indicator of the health status (mortality or morbidity), the health endpoint of interest (e.g., respiratory diseases), and the pollutant of concern.

The new conceptualization of personal exposure assessment proposed in this work provides additional insights into individual exposure to urban air pollution by providing a structured basis for the intercomparison of different methodologies. Based on the proposed structure, such comparative analysis will become more transparent and highlight the advantages and limitations of different methodologies when addressing specific research objectives.

Until now, several efforts on characterizing the spatial and temporal distributions of air pollution have been expended, but much work remains in understanding the role of individual mobility in conditioning exposures in urban areas. Efforts should also be made to refine current tools and information for modelling exposures to ambient pollutant species in all the urban microenvironments of individual’s daily routine (e.g., outdoors near home, commuting microenvironments, and non-residential indoor environments). Furthermore, very little has been done toward validating of such models at the level of the individual. Using more complex exposure estimates may introduce grater uncertainty into resultant effects. Thus, information from available sensors can be combined with personal monitoring data in order to evaluate and/or modify our current exposure models in order to reduce uncertainty in health impact assessment as well as differencing these effects from other sources of urban air pollution that lead to personal exposure.

## Figures and Tables

**Table 1:** Main characteristics of studies included in the review organized under the new classification criteria.

Reference (First Author, Year) [Ref]	Study Area	Characterisation of Air Quality	Characterisation of Individual’s Activities	Air Pollutants Analysed	Target Group
**Point-fixed and uniform exposure approach**					
Miller et al., 2007 [68]	36 U.S. Metropolitan Statistical Areas, USA	Nearest monitoring station (within 48 km)	Residential ZIP Codes	PM_2.5_	Women
Moshammer et al., 2006 [74]	Linz, Austria	One central monitoring station	School address	PM_10_ and NO_2_	Children
Laden et al., 2006 [75]	Six Cities, USA	Nearest monitoring station	Residential ZIP Codes	PM_2.5_	Adults
Schikowski et al., 2007 [76]	6 urban areas, Germany	Central background monitoring stations	Residential address	PM_10_ and NO_2_	Women
Chuang et al., 2007 [77]	Taipei, Taiwan	One central monitoring station	School address	PM_10_, O_3_, SO_2_, NO_2_, and CO	College students
Zeger et al., 2008 [78]	USA	Central monitoring stations (within 6 miles of ZIP code centroids)	Residential ZIP Codes	PM_2.5_	Elderly
Andersen et al., 2008 [79]	Copenhagen, Denmark	One central background monitoring station	Residential address	PM_10_, SO_2_, NO_2_, NO_x_, CO	Children
Pope et al., 2009 [80]	51 U.S. metropolitan areas, USA	Nearest monitoring station	Residential ZIP Codes	PM_2.5_	Adults
Belleudi et al., 2010 [81]	Rome, Itally	One central monitoring station	Residential address	PM_2.5_ and PM_10_	Adults
Collart et al., 2014 [82]	Charleroi, Belgium	Averaged pollution data (4 monitoring stations)	Residential address	PM_10_, O_3_, and NO_2_	Adults
Gao et al., 2015 [83]	Hong Kong, China	Nearest monitoring station (within 1 km)	School address	PM_10_, SO_2_, NO_2_ and O_3_	Children
**Personal exposure assessment based on point-fixed activities and variable air quality**					
Krämer et al., 2009 [87]	Small-town areas, Germany	LUR	Residential address	PM_2.5_ and NO_2_	Children
Fernández-Somoano et al., 2011 [88]	Asturias, Spain	LUR	Residential address	NO_2_ and benzene	Pregnant women
Liu et al., 2012 [89]	Eight urban areas, Switzerland	LUR	Residential address	NO_2_	Adults
Montagne et al., 2013 [90]	Utrecht, The Netherlands; Barcelona, Spain; and Helsinki, Finland	LUR	Residential address School address	PM_2.5_, Soot, NO_x_ and NO_2_	Elderly Children
Montagne et al., 2014 [91]	Utrecht, The Netherlands; Barcelona, Spain; and Helsinki, Finland	LUR	Residential address School address	Cu, Zn, Fe, K, Ni, V, Si and S	Elderly Children
Montagne et al., 2014 [92]	Utrecht, The Netherlands; Barcelona, Spain; and Helsinki, Finland	LUR	Residential address School address	Cu, Fe, K, Ni, S, Si, V and Zn	Elderly Children
Emaus et al., 2014 [93]	Utrecht, The Netherlands	LUR	Residential address	NO_x_, NO_2_, PM_10_ and PM_2.5_	Women
De Prins et al., 2014 [94]	Antwerp, Belgium	LUR	Residential address School address	BC	Children
Rosenlund et al., 2006 [95]	Stockholm, Sweden	Air dispersion modelling (100 × 100 m)	Residential address	NO_x_, NO_2_, CO, PM_2.5_ and PM_10_	Adults
Willers et al., 2013 [96]	100 cities, Sweden	Gaussian air quality dispersion model (1 × 1 km grid)	Residential address	PM_10_	Adults
Batterman et al., 2014 [97]	Detroit, USA	Gaussian air quality dispersion model	Residential address	PM_2.5_	Children
Korek et al., 2015 [98]	Stockholm, Sweden	Gaussian air quality dispersion model (25 × 25 grid cells)	Residential address	NO_x_ and PM_10_	Adults
Portnov et al., 2012 [99]	Haifa, Israel	Kriging interpolation method	Residential address	NO_2_ and PM_10_	Children
**Personal exposure assessment based on trajectory and uniform air quality**					
Lane et al., 2015 [55]	Somerville, Massachusetts, USA	Regression model	TADs	UFP	Adults
Shaddick et al., 2008 [101]	Greater London, United Kingdom	pCNEM model/nearest network monitoring station	Time–activity database (National Human Activity Pattern Survey and a 24 h recall survey)	PM_10_	Seniors
Physick et al., 2011 [102]	Melbourne, Australia	Nearest network monitoring station	TADs	NO_2_	Adults
Sarigiannis et al., 2014 [103]	Thessaloniki, Greece	Nearest monitoring station	Time–activity database	PM_2.5_ and PM_10_	Adults
**Personal exposure assessment based on trajectory and variable air quality**					
Hinwood et al., 2007 [20]	Four urban areas, Australia	Passive personal exposure monitor	TADs	BTEX	Individuals in general
Dons et al., 2011 [22]	Belgium	Active personal exposure monitor	GPS	BC	Adults
Deffner et al., 2016 [24]	Augsburg, Germany	Portable air samplers	TADs	UFP	Individuals in general
Nieuwenhuijsen et al., 2015 [28]	Barcelona, Spain	Low-cost monitors and LUR	GPS (Smart phones)	BC	Children
Özkaynak et al., 2008 [86]	USA	Exposure model (HAPEM)/Air quality modelling	Time-activity database	HAPs	Adults
Molnár et al., 2006 [104]	Göteborg, Sweden	Active personal exposure monitor	TADs	PM_1_ and PM_2.5_	Adults in general
Edwards et al., 2006 [105]	Four European cities: Athens, Helsinki, Oxford and Prague	Active personal exposure monitor	TADs	VOC	Active working age adults
Van Roosbroeck et al., 2006 [106]	Amsterdam, The Netherlands	Active personal exposure monitor	TADs	NO_x_ and PM_2.5_	School Children
Zhao et al., 2007 [107]	Denver, Colorado, USA	Active personal exposure monitor	TADs	PM_2.5_	School Children
Tang et al., 2007 [108]	Sin-Chung, Taiwan	Portable particle monitor	TADs	PM_2.5_ and PM_10_	Asthmatic children
Adgate et al., 2007 [109]	Minneapolis-St. Paul, USA	Inertial impactor environmental monitoring inlets	TADs	PM_2.5_	Individuals in general
Johannesson et al., 2007 [110]	Gothenburg, Sweden	Active personal exposure monitor	TADs	PM_1_ and PM_2.5_	Adults
Arhami et al., 2009 [111]	Four communities, Los Angeles, USA	Personal environmental monitors	Not Available	OC, EC, O_3_, NO, NO_2_, NO_x_, PM_0.25_, PM_2.5_ and PM_10_	Seniors
Du et al., 2010 [112]	Beijing, China	Active personal exposure monitor	TADs	PM_2.5_	Children and active adults
Yazar et al., 2011 [113]	Stockholm, Sweden	Passive personal exposure monitor	TADs	Benzene, 1,3-butadiene, benz(a)pyrene, NO_x_ and NO_2_	Adults
Johannesson et al., 2011 [114]	Gothenburg, Sweden	Active personal exposure monitor	TADs	PM_2.5_ and BC	Adults
Zhu et al., 2011 [115]	Camden, New Jersey, USA	Active personal exposure monitor	NA	PAH	Adults and children
Bellander et al., 2012 [116]	Stockholm, Sweden	Passive personal exposure monitor	TADs	NO_2_	Adults
Du et al., 2012 [117]	Beijing, China	Active personal exposure monitor	TADs	PM_2.5_ and NO_x_	Adults and children
Fan et al., 2012 [118]	Camden, New Jersey, USA	Passive personal exposure monitor	TADs	VOC	Socio-economically disadvantaged adults
Dadvand et al., 2012 [119]	Barcelona, Spain	Passive personal exposure monitor	TADs	PM_2.5_ and NO_x_	Pregnant women
Minguillón et al., 2012 [120]	Barcelona, Spain	Active personal exposure monitor	TADs	PM_2.5_	Pregnant women
Jahn et al., 2013 [121]	Guangzhou, China	Active personal exposure monitor	TADs	PM_2.5_	Individuals in general
Stevens et al., 2014 [122]	Detroit, USA	Active personal exposure monitor	TADs	PM_2.5_	Adults
Hinwood et al., 2014 [123]	Perth, Australia	Active personal exposure monitor	TADs	PM_2.5_	Children
Mehta et al., 2014 [124]	Ho Chi Minh, Vietnmam	Active and passive air samplers	TADs	PM_2.5_, PM_10_ and NO_2_	Children from high and low socioeconomic groups
Gatto et al., 2014 [125]	Rome, Italy	Portable air samplers	TADs	PAHs and PM_2.5_	Children Elders
Ouidir et al., 2015 [126]	Grenoble, France	Passive air samplers Air quality modelling (10 × 10 m)	GPS	PM_2.5_ and NO_2_	Pregnant women
Lei et al., 2016 [127]	Shanghai, China	Passive air samplers	GPS and TADs	PM2.5 and BC	Graduate students
Buonanno et al., 2013 [128]	Cassino, Italy	Particle counter and BC monitor	GPS and TADs	UFP and BC	Children
McNabola et al., 2011 [129]	Dublin, Ireland	Handled particle counter	GPS	PM_10_	Active adults
Huttunen et al., 2012 [130]	Kotka, Finland	Portable photometers	NA	PM_2.5_	Seniors
Buonanno et al., 2012 [131]	Cassino, Italy	Portable UFP counters	GPS and TADs	UFP	Children
Gu et al., 2015 [132]	Augsburg, Germany	Portable condensation particle counter model	TADs	UFP and PNC	Adults
Steinle et al., 2015 [133]	Edinburgh, Scotland	Low-cost monitors	GPS and TADs	PM_2.5_	Individuals in general
Jensen, 2006 [134]	Copenhagen, Denmark	Exposure model (AIRGIS)/Air pollution dispersion model	Residential and workplace address and GPS	NO_2_	Adults
Sahsuvaroglu et al., 2009 [135]	Hamilton, Canada	LUR	TADs	NO_x_ and O_3_	Seniors
Mölter et al., 2012 [136]	Greater Manchester, United Kingdom	Exposure model (MEEM)/LUR	TADs	NO_2_	Children
Gerharz et al., 2013 [137]	Münster, Germany	Lagrangian air pollution dispersion model	GPS and TADs	PM_10_	Individuals in general
Dias and Tchepel et al., 2014 [138]	Leiria, Portugal	Exposure model (ExPOSITION)/Air dispersion modelling	GPS (Smart phones)	PM_2.5_	Adults
Dons et al., 2014 [139]	Flanders, Belgium	Exposure model (AB^2^C)/LUR	TADs	BC	Adults
Tchepel et al., 2014 [140]	Leiria, Portugal	Exposure model (ExPOSITION)/Lagrangian air pollution dispersion model	GPS (Smart phones)	Benzene	Adults
Smith et al., 2016 [141]	London, United Kingdom	Exposure model (LHEM)/Air dispersion modelling	Time-activity database	NO_2_ and PM_2.5_	Adults
Su et al., 2015 [142]	California, USA	LUR	GPS (Smart phones)	NO_x_	

Note: Black carbon (BC); BTEX (benzene, toluene, ethylbenzene and xylenes); copper (Cu); elemental carbon (EC); HAPs (hazardous air pollutants); iron (Fe); land use regression models (LUR); nickel (Ni); nitrogen oxides (NO, NO_2_, NO_x_); organic carbon (OC); ozone (O_3_); particle number concentrations (PNC); particulate matter with an aerodynamic diameter smaller than respectively 10, 2.5, 1 and 0.25 μm (PM_10_, PM_2.5_, PM_1_ and PM_0.25_); potassium (K); silicon (Si); sulfur (S); sulfur dioxide (SO_2_); time-activity diaries (TADs); ultrafine particles (UFP); vanadium (V); volatile organic compounds (VOC); zinc (Zn).

## References

[1] World Health Organization (WHO) (2014). Air Quality and Health. http://www.who.int/mediacentre/factsheets/fs313/en/.

[2] World Health Organization (WHO) (2016). Ambient Air Pollution: A Global Assessment of Exposure and Burden of Disease.

[3] European Environment Agency (EEA) (2017). Air Quality in Europe—2017 Report EEA Report No 13/2017.

[4] Organization for Economic Co-Operation and Development (OECD) (2012). Environmental Outlook to 2050: The Consequences of Inaction.

[5] World Health Organization (WHO) (2013). Health Risks of Air Pollution in Europe—HRAPIE Project, Regional Office for Europe.

[6] World Health Organization (WHO) (2013). Outdoor Air Pollution a Leading Environmental Cause of Cancer Deaths.

[7] Enstrom J.E. (2017). Fine particulate matter and total mortality in cancer prevention study cohort reanalysis. Dose Response.

[8] Szpiro A.A., Sampson P.D., Sheppard L., Lumley T., Adar S.D., Kaufman J. (2008). Predicting Intra-Urban Variation in Air Pollution Concentrations with Complex Spatio-Temporal Interactions.

[9] Peng R.D., Bell M.L. (2010). Spatial misalignment in time series studies of air pollution and health data. Biostatistics.

[10] Zartarian V.G., Bahadori T., McKone T.E. (2004). Feature Article: The Adoption of an Official ISEA Glossary. J. Expo. Anal. Environ. Epidemiol..

[11] World Health Organization (WHO) (2004). Occupational Carcinogens: Assessing the Environmental Burden of Disease at National and Local Levels.

[12] Van Leeuwen C.J., Vermeire T.G. (2007). Risk Assessment of Chemicals: An Introduction.

[13] International Programme on Chemical Safety (IPCS) (2009). Principles for Modelling Dose-Response for the Risk Assessment of Chemicals.

[14] Lisella F.S. (1994). The VNR Dictionary of Environmental Health and Safety.

[15] Georgopolous P.G., Lioy P.J. (1994). Conceptual and Theoretical Aspects of Human Exposure and Dose Assessment. J. Expo. Anal. Environ. Epidemiol..

[16] World Health Organisation (WHO) (1999). Monitoring Ambient Air Quality for Health Impact Assessment.

[17] US Environmental Protection Agency (USEPA) (2005). Guidelines for Carcinogen Risk Assessment (EPA/630/P-03/001B).

[18] White E., Armstrong B.K., Saracci R., Armstrong B.K. (2008). Principles of Exposure Measurement in Epidemiology: Collecting, Evaluating, and Improving Measures of Disease Risk Factors.

[19] Monn C. (2001). Exposure assessment of air pollutants: A review on spatial heterogeneity and indoor/outdoor/personal exposure to suspended particulate matter, nitrogen dioxide and ozone. Atmos. Environ..

[20] Hinwood A.L., Rodriguez C., Runnion T., Farrar D., Murray F., Horton A., Whitworth T. (2007). Risk factors for increased BTEX exposure in four Australian cities. Chemosphere.

[21] Branis M., Lazaridis M., Colbeck I. (2010). Personal exposure measurements. Human Exposure to Pollutants via Dermal Absorption and Inhalation.

[22] Dons E., Panis L.I., Van Poppel M., Theunis J., Willems H., Torfs R., Wets G. (2011). Impact of time–activity patterns on personal exposure to black carbon. Atmos. Environ..

[23] Özkaynak H., Baxter L.K., Dionisio K.L., Burke J. (2013). Air pollution exposure prediction approaches used in air pollution epidemiology studies. J. Exp. Sci. Environ. Epidemiol..

[24] Deffner V., Küchenhoff H., Maier V., Pitz M., Cyrys J., Breitner S., Peters A. (2016). Personal exposure to ultrafine particles: Two-level statistical modeling of background exposure and time-activity patterns during three seasons. J. Exp. Sci. Environ. Epidemiol..

[25] Nazaroff W.W., Singer B.C. (2004). Inhalation of hazardous air pollutants from environmental tobacco smoke in US residences. J. Exp. Sci. Environ. Epidemiol..

[26] Darrow L.A., Klein M., Sarnat J.A., Mulholland J.A., Strickland M.J., Sarnat S.E., Tolbert P.E. (2011). The use of alternative pollutant metrics in time-series studies of ambient air pollution and respiratory emergency department visits. J. Exp. Sci. Environ. Epidemiol..

[27] Zhang J.J., Lioy P.J. (2002). Human exposure assessment in air pollution systems. Sci. World J..

[28] Nieuwenhuijsen M.J., Donaire-gonzalez D., Rivas I., Cirach M., Hoek G., Seto E., Jerrett M., Sunyer J. (2015). Variability in and agreement between modelled and personal continuously measured black carbon levels using novel smartphone and sensor technologies. Environ. Sci. Technol..

[29] Nieuwenhuijsen M.J. (2015). Exposure Assessment in Environmental Epidemiology.

[30] Zanetti P. (2003). Air Quality Modeling—Theories, Methodologies, Computational Techniques, and Available Databases and Software.

[31] Wilson J.G., Zawar-Reza P. (2006). Intraurban-scale dispersion modelling of particulate matter concentrations: Applications for exposure estimates in cohort studies. Atmos. Environ..

[32] Gulliver J., Briggs D.J. (2005). Time-space modeling of journey-time exposure to traffic-related air pollution using GIS. Environ. Res..

[33] Georgopoulos P., Isukapalli S., Burke J., Napelenok S., Palma T., Langstaff J., Majeed M., He S., Byun D., Cohen M. (2009). Air quality modelling needs for exposure assessment from the source-to-outcome perspective. Environ. Manag..

[34] HEI (Health Effects Institute) (2010). Traffic-Related Air Pollution: A Critical Review of the Literature on Emissions, Exposure, and Health Effects.

[35] Son J.Y., Bell M.L., Lee J.T. (2010). Individual exposure to air pollution and lung function in Korea Spatial analysis using multiple exposure approaches. Environ. Res..

[36] Briggs D.J. (2008). A framework for integrated environmental health impact assessment of systemic risks. Environ. Health.

[37] De Nazelle A., Nieuwenhuijsen M.J., Anto J.M., Brauer M., Briggs D., BraunFahrlander C., Cavill N., Cooper A.R., Desqueyroux H., Fruin S. (2011). Improving health through policies that promote active travel: A review of evidence to support integrated health impact assessment. Environ. Int..

[38] Hoek G., Krishnan R.M., Beelen R., Peters A., Ostro B., Brunekreef B., Kaufman J.D. (2013). Long-term air pollution exposure and cardio-respiratory mortality: A review. Environ. Health.

[39] Nikolova I., Janssen S., Vrancken K., Vos P., Mishra V., Berghmans P. (2011). Size Resolved Ultrafine Particles Emission Model—A Continuous Size Distribution Approach. Sci. Total Environ..

[40] Dionisio K.L., Isakov V., Baxter L.K., Sarnat J.A., Sarnat S.E., Burke J., Rosenbaum A., Graham S.E., Cook R., Mulholland J. (2013). Development and evaluation of alternative approaches for exposure assessment of multiple air pollutants in Atlanta, Georgia. J. Expo. Sci. Environ. Epidemiol..

[41] Zou B., Wilson J.G., Zhan F.B., Zeng Y. (2009). Air pollution exposure assessment methods utilized in epidemiological studies. J. Environ. Monit..

[42] Colbeck I., Nasir Z.A., Lazaridis M., Colbeck I. (2010). Indoor air pollution. Human Exposure to Pollutants via Dermal Absorption and Inhalation, 17.

[43] Brunekreef B., Janssen N.A.H., de Hartog J.J., Oldenwening M., Meliefste K., Hoek G., Lanki T., Timonen K.L., Vallius M., Pekkanen J. (2005). Personal, Indoor, and Outdoor Exposures to PM_2.5_ and Its Components for Groups of Cardiovascular Patients in Amsterdam and Helsinki.

[44] Koutrakis P., Suh H.H., Sarnat J.A., Brown K.W., Coull B.A., Schwartz J. (2005). Characterization of Particulate and Gas Exposures of Sensitive Subpopulations Living in Baltimore and Boston.

[45] EC (European Commission) (2005). HEXPOC Human Exposure Characterisation of Chemical Substances, Quantification of Exposure Routes.

[46] Shuai J., Yang W., Ahn H., Kim S., Lee S., Yoon S.U. (2013). Contribution of indoor and outdoor nitrogen dioxide to indoor air quality of wayside shops. J. UOEH.

[47] Wu X., Bennett D.H., Lee K., Cassady D.L., Ritz B., Hertz-Picciotto I. (2011). Longitudinal variability of time-location/activity patterns of population at different ages: A longitudinal study in California. Environ. Health.

[48] Batty M. (2009). Cities as Complex Systems: Scaling, Interactions, Networks, Dynamics and Urban Morphologies.

[49] Portugali J., Meyer H., Stolk E., Tan E. (2012). Complexity Theories of Cities Have Come of Age: An Overview with Implications to Urban Planning and Design.

[50] World Health Organization (WHO) (2005). Principles of Characterizing and Applying Human Exposure Models.

[51] McKone T.E., Ryan P.B., Ozkaynak H. (2008). Exposure information in environmental health research: Current opportunities and future directions for particulate matter, ozone, and toxic air pollutants. J. Exp. Sci. Environ. Epidemiol..

[52] Beckx C., Int Panis L., Arentze T., Janssens D., Torfs R., Broekx S., Wets G. (2009). A dynamic activity-based population modelling approach to evaluate exposure to air pollution: Methods and application to a Dutch urban area. Environ. Impact Assess. Rev..

[53] Zou B., Wilson J.G., Zhan F.B., Zeng Y. (2009). Spatially differentiated and source-specific population exposure to ambient urban air pollution. Atmos. Environ..

[54] Lane K.J., Scammell M.K., Levy J.I., Fuller C.H., Parambi R., Zamore W., Brugge D. (2013). Positional error and time-activity patterns in near-highway proximity studies: An exposure misclassification analysis. Environ. Health.

[55] Lane K.J., Levy J.I., Scammell M.K., Patton A.P., Durant J.L., Mwamburi M., Brugge D. (2015). Effect of time-activity adjustment on exposure assessment for traffic-related ultrafine particles. J. Exp. Sci. Environ. Epidemiol..

[56] Wallace L., Nelson W., Ziegenfus R., Pellizzari E., Michael L., Whitmore R., Zelon H., Hartwell T., Perritt R., Westerdahl D. (1991). The Los Angeles TEAM study: Personal exposures, indoor-outdoor air concentrations, and breath concentrations of 25 volatile organic compounds. J. Exp. Anal. Environ. Epidemiol..

[57] Klepeis N.E., Nelson W.C., Ott W.R., Robinson J.P., Tsang A.M., Switzer P., Behar J.V., Hern S.C. (2001). The National Human Activity Pattern Survey (NHAPS): A resource for assessing exposure to environmental pollutants. J. Expo. Anal. Environ. Epidemiol..

[58] Ballesta P.P., Field R.A., Connolly R., Cao N., Caracena A.B., De Saeger E. (2006). Population exposure to benzene: One day cross-sections in six European cities. Atmos. Environ..

[59] Rainham D., McDowell I., Krewski D., Sawada M. (2010). Conceptualizing the healthscape: Contributions of time geography, location technologies and spatial ecology to place and health research. Soc. Sci. Med..

[60] Lawless P., Thornburg J., Rodes C., Vette A., Williams R. (2012). Personal exposure monitoring protocol compliance: Quantitative measurement. J. Expo. Sci. Environ. Epidemiol..

[61] Van Ryswyk K., Wheeler A.J., Wallace L., Kearney J., You H., Kulka R., Xu X. (2014). Impact of microenvironments and personal activities on personal PM_2.5_ exposures among asthmatic children. J. Expo. Sci. Environ. Epidemiol..

[62] Gonzalez M.C., Hidalgo C.A., Barabasi A.L. (2008). Understanding human mobility patterns. Nature.

[63] Song C., Qu Z., Blumm N., Barabási A.L. (2010). Limits of predictability in human mobility. Science.

[64] Chaix B., Méline J., Duncan S., Merrien C., Karusisi N., Perchoux C., Lewin A., Labadi K., Kestens Y. (2013). GPS tracking in neighborhood and health studies: A step forward for environmental exposure assessment, a step backward for causal inference?. Health Place.

[65] Wu J., Jiang C., Liu Z., Houston D., Jaimes G., McConnell R. (2010). Performances of Different Global Positioning System Devices for Time-Location Tracking in Air Pollution Epidemiological Studies. J. Environ. Health Insights.

[66] Zheng Y., Zhou X. (2011). Computing with Spatial Trajectories.

[67] USEPA (US Environmental Protection Agency) (1992). Guidelines for Exposure Assessment (EPA/600/Z-92/001). Environmental Protection Agency.

[68] Miller K.A., Siscovick D.S., Sheppard L., Shepherd K., Sullivan J.H., Anderson G.L., Kaufman J.D. (2007). Long-term exposure to air pollution and incidence of cardiovascular events in women. N. Engl. J. Med..

[69] Matthews S.A., Burton S.P., Kemp M., Leung S.A., Matthews D.T., Takeuchi L.M. (2011). Spatial polygamy and the heterogeneity of place: Studying people and place via egocentric methods. Communities, Neighborhoods, and Health: Expanding the Boundaries of Place.

[70] Health Effects Institute (2015). HEI Strategic Plan for Understanding the Health Effects of Air Pollution 2015–2020.

[71] Kwan M.P. (2009). From place-based to people-based exposure measures. Soc. Sci. Med..

[72] Fang T.B., Lu Y. (2012). Personal real-time air pollution exposure assessment methods promoted by information technological advances. Ann. GIS.

[73] Steinle S., Reis S., Sabel C.E. (2013). Quantifying human exposure to air pollution—Moving from static monitoring to spatio-temporally resolved personal exposure assessment. Sci. Total Environ..

[74] Moshammer H., Hutter H.P., Hauck H., Neuberger M. (2006). Low levels of air pollution induce changes of lung function in a panel of schoolchildren. Eur. Respir. J..

[75] Laden F., Schwartz J., Speizer F.E., Dockery D.W. (2006). Reduction in fine particulate air pollution and mortality: Extended follow-up of the Harvard Six Cities study. Am. J. Respir. Crit. Care Med..

[76] Schikowski T., Sugiri D., Ranft U., Gehring U., Heinrich J., Wichmann H.E., Krämer U. (2007). Does respiratory health contribute to the effects of long-term air pollution exposure on cardiovascular mortality?. Respir. Res..

[77] Chuang K.J., Chan C.C., Su T.C., Lee C.T., Tang C.S. (2007). The effect of urban air pollution on inflammation, oxidative stress, coagulation, and autonomic dysfunction in young adults. Am. J. Respir. Crit. Care Med..

[78] Zeger S.L., Dominici F., McDermott A., Samet J.M. (2008). Mortality in the Medicare population and chronic exposure to fine particulate air pollution in urban centers (2000–2005). Environ. Health Perspect..

[79] Andersen Z.J., Loft S., Ketzel M., Stage M., Scheike T., Hermansen M.N., Bisgaard H. (2008). Ambient air pollution triggers wheezing symptoms in infants. Thorax.

[80] Pope C.A., Ezzati M., Dockery D.W. (2009). Fine-Particulate Air Pollution and Life Expectancy in the United States. N. Engl. J. Med..

[81] Belleudi V., Faustini A., Stafoggia M., Cattani G., Marconi A., Perucci C.A., Forastiere F. (2010). Impact of fine and ultrafine particles on emergency hospital admissions for cardiac and respiratory diseases. Epidemiology.

[82] Collart P., Coppieters Y., Mercier G., Massamba Kubuta V., Leveque A. (2014). Comparison of four case-crossover study designs to analyze the association between air pollution exposure and acute myocardial infarction. Int. J. Environ. Health Res..

[83] Gao Y., Chan E.Y., Li L., Lau P.W., Wong T.W. (2015). Chronic effects of ambient air pollution on respiratory morbidities among Chinese children: A cross-sectional study in Hong Kong. BMC Public Health.

[84] Baklanov A., Hänninen O., Slordal L.H., Kukkonen J., Bjergene N., Fay B., Finardi S., Hoe S.C., Jantunen M., Karppinen A. (2007). Integrated systems for forecasting urban meteorology, air pollution and population exposure. Atmos. Chem. Phys..

[85] Nethery E., Leckie S.E., Teschke K., Brauer M. (2008). From measures to models: An evaluation of air pollution exposure assessment for epidemiological studies of pregnant women. Occup. Environ. Med..

[86] Özkaynak H., Palma T., Touma J.S., Thurman J. (2008). Modeling population exposures to outdoor sources of hazardous air pollutants. J. Expo. Sci. Environ. Epidemiol..

[87] Krämer U., Sugiri D., Ranft U., Krutmann J., von Berg A., Berdel D., Heinrich J. (2009). Eczema, respiratory allergies, and traffic-related air pollution in birth cohorts from small-town areas. J. Dermatol. Sci..

[88] Fernández-Somoano A., Estarlich M., Ballester F., Fernández-Patier R., Aguirre-Alfaro A., Herce-Garraleta M.D., Tardón A. (2011). Outdoor NO_2_ and benzene exposure in the INMA (Environment and Childhood) Asturias cohort (Spain). Atmos. Environ..

[89] Liu L.J.S., Tsai M.Y., Keidel D., Gemperli A., Ineichen A., Hazenkamp-von Arx M., Straehl P. (2012). Long-term exposure models for traffic related NO_2_ across geographically diverse areas over separate years. Atmos. Environ..

[90] Montagne D., Hoek G., Nieuwenhuijsen M., Lanki T., Pennanen A., Portella M., Brunekreef B. (2013). Agreement of land use regression models with personal exposure measurements of particulate matter and nitrogen oxides air pollution. Environ. Sci. Technol..

[91] Montagne D., Hoek G., Nieuwenhuijsen M., Lanki T., Siponen T., Portella M., Brunekreef B. (2014). Temporal associations of ambient PM_2.5_ elemental concentrations with indoor and personal concentrations. Atmos. Environ..

[92] Montagne D., Hoek G., Nieuwenhuijsen M., Lanki T., Pennanen A., Portella M., Cirach M. (2014). The association of LUR modeled PM_2.5_ elemental composition with personal exposure. Sci. Total Environ..

[93] Emaus M.J., Bakker M.F., Beelen R.M., Veldhuis W.B., Peeters P.H., van Gils C.H. (2014). Degree of urbanization and mammographic density in Dutch breast cancer screening participants: Results from the EPIC-NL cohort. Breast Cancer Res. Treat..

[94] De Prins S., Dons E., Van Poppel M., Panis L.I., Van de Mieroop E., Nelen V., Koppen G. (2014). Airway oxidative stress and inflammation markers in exhaled breath from children are linked with exposure to black carbon. Environ. Int..

[95] Rosenlund M., Berglind N., Pershagen G., Hallqvist J., Jonson T., Bellander T. (2006). Long-term exposure to urban air pollution and myocardial infarction. Epidemiology.

[96] Willers S.M., Eriksson C., Gidhagen L., Nilsson M.E., Pershagen G., Bellander T. (2013). Fine and coarse particulate air pollution in relation to respiratory health in Sweden. Eur. Respir. J..

[97] Batterman S., Burke J., Isakov V., Lewis T., Mukherjee B., Robins T. (2014). A comparison of exposure metrics for traffic-related air pollutants: Application to epidemiology studies in Detroit, Michigan. Int. J. Environ. Res. Public Health.

[98] Korek M.J., Bellander T.D., Lind T., Bottai M., Eneroth K.M., Caracciolo B., Magnusson P.K. (2015). Traffic-related air pollution exposure and incidence of stroke in four cohorts from Stockholm. J. Expo. Sci. Environ. Epidemiol..

[99] Portnov B.A., Reiser B., Karkabi K., Cohen-Kastel O., Dubnov J. (2012). High prevalence of childhood asthma in Northern Israel is linked to air pollution by particulate matter: Evidence from GIS analysis and Bayesian Model Averaging. Int. J. Environ. Health Res..

[100] Zou B. (2010). How should environmental exposure risk be assessed? A comparison of four methods for exposure assessment of air pollutions. Environ. Monit. Assess..

[101] Shaddick G., Lee D., Zidek J.V., Salway R. (2008). Estimating exposure response functions using ambient pollution concentrations. Ann. Appl. Stat..

[102] Physick W., Powell J., Cope M., Boast K., Lee S. (2011). Measurements of personal exposure to NO_2_ and modelling using ambient concentrations and activity data. Atmos. Environ..

[103] Sarigiannis D.Α., Karakitsios S.P., Kermenidou M., Nikolaki S., Zikopoulos D., Semelidis S., Tzimou R. (2014). Total exposure to airborne particulate matter in cities: The effect of biomass combustion. Sci. Total Environ..

[104] Molnár P., Johannesson S., Boman J., Barregård L., Sällsten G. (2006). Personal exposures and indoor, residential outdoor, and urban background levels of fine particle trace elements in the general population. J. Environ. Monit..

[105] Edwards R.D., Schweizer C., Llacqua V., Lai H.K., Jantunen M., Bayer-Oglesby L., Künzli N. (2006). Time–activity relationships to VOC personal exposure factors. Atmos. Environ..

[106] Van Roosbroeck S., Wichmann J., Janssen N.A., Hoek G., van Wijnen J.H., Lebret E., Brunekreef B. (2006). Long-term personal exposure to traffic-related air pollution among school children, a validation study. Sci. Total Environ..

[107] Zhao W., Hopke P.K., Gelfand E.W., Rabinovitch N. (2007). Use of an expanded receptor model for personal exposure analysis in schoolchildren with asthma. Atmos. Environ..

[108] Tang C.S., Chang L.T., Lee H.C., Chan C.C. (2007). Effects of personal particulate matter on peak expiratory flow rate of asthmatic children. Sci. Total Environ..

[109] Adgate J.L., Mongin S.J., Pratt G.C., Zhang J., Field M.P., Ramachandran G., Sexton K. (2007). Relationships between personal, indoor, and outdoor exposures to trace elements in PM_2.5_. Sci. Total Environ..

[110] Johannesson S., Gustafson P., Molnar P., Barregard L., Sallsten G. (2007). Exposure to fine particles ([PM_2.5_] and [PM_1_]) and black smoke in the general population: Personal, indoor, and outdoor levels. J. Expo. Sci. Environ. Epidemiol..

[111] Arhami M., Polidori A., Delfino R.J., Tjoa T., Sioutas C. (2009). Associations between personal, indoor, and residential outdoor pollutant concentrations: Implications for exposure assessment to size-fractionated particulate matter. J. Air Waste Manag. Assoc..

[112] Du X., Kong Q., Ge W., Zhang S., Fu L. (2010). Characterization of personal exposure concentration of fine particles for adults and children exposed to high ambient concentrations in Beijing, China. J. Environ. Sci..

[113] Yazar M., Bellander T., Merritt A.S. (2011). Personal exposure to carcinogenic and toxic air pollutants in Stockholm, Sweden: A comparison over time. Atmos. Environ..

[114] Johannesson S., Rappaport S.M., Sallsten G. (2011). Variability of environmental exposure to fine particles, black smoke, and trace elements among a Swedish population. J. Expo. Sci. Environ. Epidemiol..

[115] Zhu X., Wu X., Jung K.H., Ohman-Strickland P., Bonanno L.J., Lioy P.J. (2011). Ambient concentrations and personal exposure to polycyclic aromatic hydrocarbons (PAH) in an urban community with mixed sources of air pollution. J. Expo. Sci. Environ. Epidemiol..

[116] Bellander T., Wichmann J., Lind T. (2012). Individual exposure to NO_2_ in relation to spatial and temporal exposure indices in Stockholm, Sweden: The INDEX study. PLoS ONE.

[117] Du X., Wu Y., Fu L., Wang S., Zhang S., Hao J. (2012). Intake fraction of PM_2.5_ and NO_X_ from vehicle emissions in Beijing based on personal exposure data. Atmos. Environ..

[118] Fan Z.T., Zhu X., Jung K.H., Ohman-Strickland P., Weisel C.P., Lioy P.J. (2012). Exposures to volatile organic compounds (VOCs) and associated health risks of socio-economically disadvantaged population in a “hot spot” in Camden, New Jersey. Atmos. Environ..

[119] Dadvand P., de Nazelle A., Triguero-Mas M., Schembari A., Cirach M., Amoly E., Nieuwenhuijsen M. (2012). Surrounding greenness and exposure to air pollution during pregnancy: An analysis of personal monitoring data. Environ. Health Perspect..

[120] Minguillón M.C., Schembari A., Triguero-Mas M., de Nazelle A., Dadvand P., Figueras F., Querol X. (2012). Source apportionment of indoor, outdoor and personal PM_2.5_ exposure of pregnant women in Barcelona, Spain. Atmos. Environ..

[121] Jahn H.J., Kraemer A., Chen X.C., Chan C.Y., Engling G., Ward T.J. (2013). Ambient and personal PM_2.5_ exposure assessment in the Chinese megacity of Guangzhou. Atmos. Environ..

[122] Stevens C., Williams R., Jones P. (2014). Progress on understanding spatial and temporal variability of PM_2.5_ and its components in the Detroit Exposure and Aerosol Research Study (DEARS). Environ. Sci. Process. Impacts.

[123] Hinwood A., Callan A.C., Heyworth J., McCafferty P., Sly P.D. (2014). Children’s personal exposure to PM_10_ and associated metals in urban, rural and mining activity areas. Chemosphere.

[124] Mehta S., Sbihi H., Dinh T.N., Xuan D.V., Thanh L.L.T., Thanh C.T., Brauer M. (2014). Effect of poverty on the relationship between personal exposures and ambient concentrations of air pollutants in Ho Chi Minh City. Atmos. Environ..

[125] Gatto M.P., Gariazzo C., Gordiani A., L’Episcopo N., Gherardi M. (2014). Children and elders exposure assessment to particle-bound polycyclic aromatic hydrocarbons (PAHs) in the city of Rome, Italy. Environ. Sci. Pollut. Res..

[126] Ouidir M., Giorgis-Allemand L., Lyon-Caen S., Morelli X., Cracowski C., Pontet S., Slama R. (2015). Estimation of exposure to atmospheric pollutants during pregnancy integrating space–time activity and indoor air levels: Does it make a difference?. Environ. Int..

[127] Lei X., Xiu G., Li B., Zhang K., Zhao M. (2016). Individual exposure of graduate students to PM_2.5_ and black carbon in Shanghai, China. Environ. Sci. Pollut. Res..

[128] Buonanno G., Marini S., Morawska L., Fuoco F.C. (2012). Individual dose and exposure of Italian children to ultrafine particles. Sci. Total Environ..

[129] McNabola A., McCreddin A., Gill L.W., Broderick B.M. (2011). Analysis of the relationship between urban background air pollution concentrations and the personal exposure of office workers in Dublin, Ireland, using baseline separation techniques. Atmos. Pollut. Res..

[130] Huttunen K., Siponen T., Salonen I., Yli-Tuomi T., Aurela M., Dufva H., Peters A. (2012). Low-level exposure to ambient particulate matter is associated with systemic inflammation in ischemic heart disease patients. Environ. Res..

[131] Buonanno G., Stabile L., Morawska L., Russi A. (2013). Children exposure assessment to ultrafine particles and black carbon: The role of transport and cooking activities. Atmos. Environ..

[132] Gu J., Kraus U., Schneider A., Hampel R., Pitz M., Breitner S., Cyrys J. (2015). Personal day-time exposure to ultrafine particles in different microenvironments. Int. J. Hyg. Environ. Health.

[133] Steinle S., Reis S., Sabel C.E., Semple S., Twigg M.M., Braban C.F., Wu H. (2015). Personal exposure monitoring of PM_2.5_ in indoor and outdoor microenvironments. Sci. Total Environ..

[134] Jensen S.S. (2006). A GIS-GPS modeling system for personal exposure to traffic air pollution. Epidemiology.

[135] Sahsuvaroglu T., Su J.G., Brook J., Burnett R., Loeb M., Jerrett M. (2009). Predicting personal nitrogen dioxide exposure in an elderly population: Integrating residential indoor and outdoor measurements, fixed-site ambient pollution concentrations, modeled pollutant levels, and time–activity patterns. J. Toxicol. Environ. Health Part A.

[136] Mölter A., Lindley S., de Vocht F., Agius R., Kerry G., Johnson K., Simpson A. (2012). Performance of a microenviromental model for estimating personal NO_2_ exposure in children. Atmos. Environ..

[137] Gerharz L.E., Klemm O., Broich A.V., Pebesma E. (2013). Spatio-temporal modelling of individual exposure to air pollution and its uncertainty. Atmos. Environ..

[138] Dias D., Tchepel O. (2014). Modelling of human exposure to air pollution in the urban environment: A GPS-based approach. Environ. Sci. Pollut. Res..

[139] Dons E., Van Poppel M., Kochan B., Wets G., IntPanis L. (2014). Implementation and validation of a modeling framework to assess personal exposure to black carbon. Environ. Int..

[140] Tchepel O., Dias D., Costa C., Santos B.F., Teixeira J.P. (2014). Modeling of human exposure to benzene in urban environments. J. Toxicol. Environ. Health Part A.

[141] Smith J.D., Mitsakou C., Kitwiroon N., Barratt B.M., Walton H.A., Taylor J.G., Beevers S.D. (2016). London Hybrid exposure model: Improving human exposure estimates to NO_2_ and PM_2.5_ in an urban setting. Environ. Sci. Technol..

[142] Su J.G., Jerrett M., Meng Y.Y., Pickett M., Ritz B. (2015). Integrating smart-phone based momentary location tracking with fixed site air quality monitoring for personal exposure assessment. Sci. Total Environ..

